# Using Three-Dimensional Computer Graphics for Anomalous Drainage of the Inferior Vena Cava

**DOI:** 10.1016/j.atssr.2024.11.002

**Published:** 2024-11-13

**Authors:** Shigeto Tsuji, Yasutaka Hirata, Minoru Ono

**Affiliations:** 1 Department of Cardiovascular Surgery, The University of Tokyo, Tokyo, Japan

## Abstract

A 5-year-old girl was diagnosed with an atrial septal defect and anomalous drainage of the right lower pulmonary vein into the right atrium at the age of 2 years. On reevaluation of computed tomography findings, anomalous drainage of the inferior vena cava to the left atrium was suspected. For accurate diagnosis, we created a cardiac 3-dimensional computer graphics model based on computed tomography imaging, which clearly demonstrated an anomalous connection of the inferior vena cava to the left atrium combined with an atrial septal defect. The 3-dimensional reconstruction was useful for the accurate diagnosis of the relatively uncommon anatomy.

Anomalous drainage of the inferior vena cava (IVC) to the left atrium (LA) is a rare anomaly that leads to systemic desaturation due to a right-to-left shunt, and only a few cases have been reported.^1^, ^2^, ^3^ Although the right sinus venosus valve normally regresses and becomes the crista terminalis, eustachian valve, and thebesian valve, it is suggested that this abnormality arises as the right sinus venosus valve persists and fuses with the superior part of the secundum septum.^1^^,^^2^^,^^4^^,^^5^ Approximately half of the reported cases occur in conjunction with an atrial septal defect (ASD).^4^^,^^5^ We report a case wherein the patient was accurately diagnosed with anomalous drainage of the IVC to the LA combined with an ASD by creating a 3-dimensional computer graphics (3DCG) model of the patient’s heart.

The patient, a 5-year-old girl, weighed 16.8 kg. At 6 months of age, a cardiac murmur was pointed out, and the patient was diagnosed with ASD by echocardiography. At 2 years of age, she was referred to our institution for further assessment and treatment. By contrast-enhanced computed tomography (CT), she was diagnosed with ASD in association with anomalous drainage of the right lower pulmonary vein (RLPV) to the right atrium (RA) because the location of inflow from the IVC was considered to be the RA. There were no signs of fatigue or growth abnormalities. At 5 years of age, she was referred to our department for surgical treatment because of progressively worsening pulmonary hypertension.

On examination, the peripheral oxygen saturation was 95% on room air, and there were no clinical features such as cyanosis or clubbing of fingers. Cardiac auscultation revealed a fixed second heart sound and a systolic ejection murmur at the left upper sternal border. Chest radiography revealed a cardiothoracic ratio of 50%, with bilateral hilar pulmonary vessel enlargement. Electrocardiography revealed regular sinus rhythm with right axis deviation, RA enlargement, and right ventricular hypertrophy. Transthoracic echocardiography showed RA and ventricular dilation and an ASD approximately 20 mm in size with a left-to-right shunt located in the posteroinferior part of the septum. The inferior rim of the ASD was 6.8 mm, and the posterior rim was deficient. The tricuspid regurgitation pressure gradient was 39 mm Hg.

On reevaluation of the contrast-enhanced CT findings, the IVC was suspected to be anomalously connected to the LA. To assess the precise intracardiac anatomy, we created a cardiac 3DCG model based on Digital Imaging and Communications in Medicine (DICOM) data from contrast-enhanced CT, using the Viewtify visualization software (SCIEMENT, Inc).^6^^,^^7^ A cross-sectional cut along the RA indicated an ASD and an anomalous connection of the IVC to the LA (Figure 1 and Video). The RLPV was connected to the LA. As shown by the broken red line in Figure 1, we anticipated the suture line to redirect the blood of the IVC to the RA. Finally, the patient was diagnosed with anomalous drainage of the IVC into the LA, combined with ASD, and surgical correction was performed.

**Figure 1:**
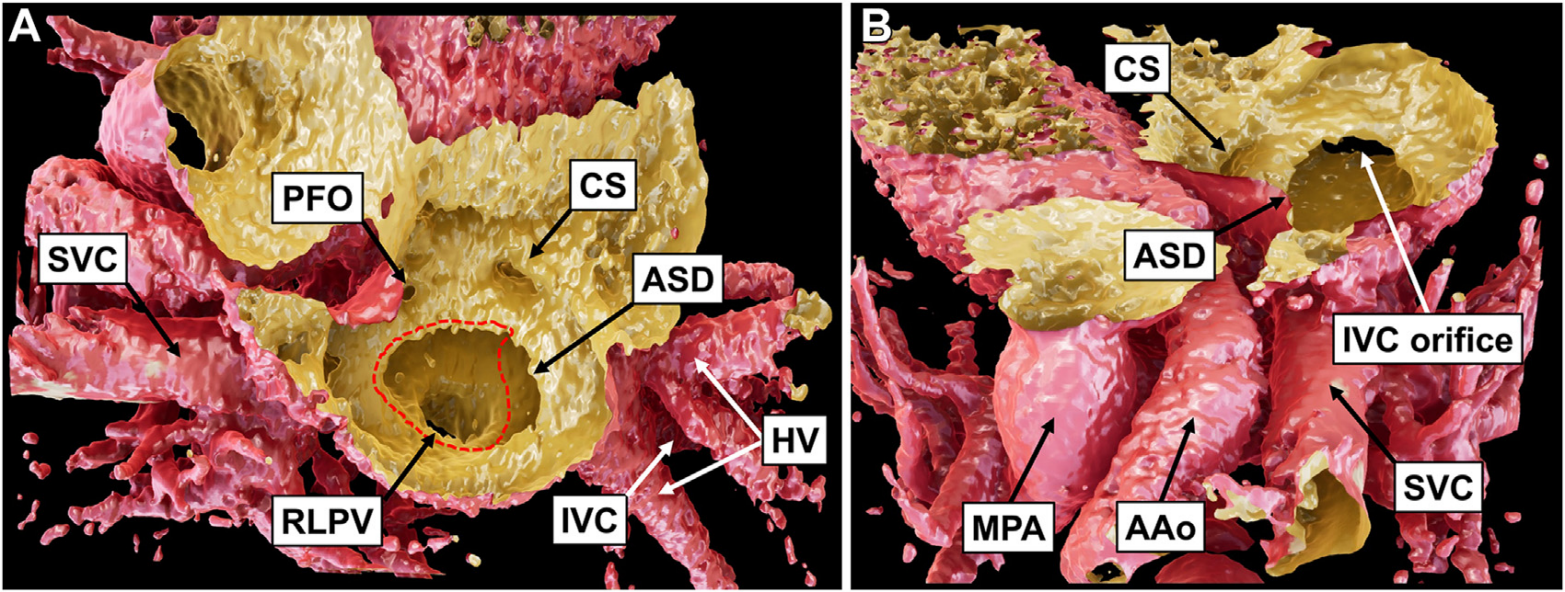
(A) A cross-sectional cut along the right atrium in the patient’s cardiac 3-dimensional computer graphics model. An atrial septal defect (ASD) and anomalous drainage of the inferior vena cava (IVC) to the left atrium are observed. The right lower pulmonary vein (RLPV) is connected to the left atrium. A broken red line demonstrates the suture line to reroute the IVC to the right atrium. (B) A cranial view clearly showing the IVC draining to the left atrium. (AAo, ascending aorta; CS, coronary sinus; HV, hepatic vein; MPA, main pulmonary artery; PFO, patent foramen ovale; SVC, superior vena cava.)

The operation was performed through a median sternotomy. Cardiopulmonary bypass was established with ascending aortic perfusion and bicaval drainage. The heart was arrested with antegrade cardioplegia. After the superior vena cava and IVC were snared, the RA was opened. An ASD was located posteroinferior to the patent foramen ovale, and the RLPV was connected to the LA (Figure 2A and Video). By releasing the snare of the IVC, the blood from the IVC was drained directly into the LA (Figure 2B and Video). Based on the suture line as anticipated preoperatively by the 3DCG model, we established a rerouting pathway of the IVC to the RA using an expanded polytetrafluoroethylene patch while taking care not to create stenosis of the IVC and RLPV. Simultaneously, the patent foramen ovale was closed.

**Figure 2:**
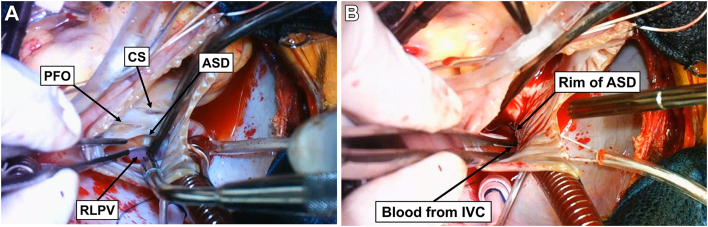
(A) Intraoperative photograph showing an atrial septal defect (ASD) located posteroinferior to the patent foramen ovale (PFO). The right lower pulmonary vein (RLPV) is connected to the left atrium. (B) Blood from the inferior vena cava (IVC) drains directly into the left atrium on releasing the snare of the IVC. (CS, coronary sinus.)

Postoperative echocardiography revealed that IVC blood drained into the RA, with no stenosis of the IVC or RLPV. The peripheral oxygen saturation increased to 100% on room air. The patient’s postoperative course was uneventful, and she was discharged on postoperative day 8.

## Comment

In cases in which anomalous connection of the IVC to the LA in association with ASD is suspected, contrast echocardiography and selective IVC angiography have been reported to be useful.^1^, ^2^, ^3^, ^4^, ^5^ Although injection of contrast medium from the arms demonstrates normal opacification of the RA, the contrast agent when injected from the lower legs shows opacification of the LA, followed by RA opacification through the ASD. Although anomalous drainage of the IVC to the LA often causes cyanosis or clubbing of fingers, cases in which oxygen desaturation remains mild have also been reported.^5^ In this reported case, the absence of evident cyanosis contributed to a delayed diagnosis. On reevaluation of the CT scan immediately preoperatively, anomalous drainage of the IVC was suspected for the first time.

For an accurate diagnosis, we used the Viewtify visualization software. By importing the DICOM data of contrast-enhanced CT into the software, a cardiac 3DCG model can be created. Although 3-dimensional model printing typically takes hours or even days, this software can generate a 3DCG model within a few minutes after importing the data. The intimal lumen of the heart can be distinguished from the cardiac walls by use of image thresholding. Unlike conventional 3DCG reconstructions of CT images or 3-dimensional printed models, this software can generate a cross section from any angle in real time. In our patient, by observing the heart at the RA cross section, we could accurately comprehend the spatial relationship between the IVC, RLPV, and ASD preoperatively and successfully redirect blood from the IVC to the RA. A cardiac 3DCG model created by use of this software is an effective tool for understanding the intracardiac structure of complicated congenital heart diseases and for simulating a correct rerouting course preoperatively.
